# Supplementary material for: Macrocyclization of linear molecules by deep learning to facilitate macrocyclic drug candidates discovery
Source: Nat Commun. 2023 Jul 28;14:4552. doi: 10.1038/s41467-023-40219-8 (PMC10382584; doi:10.1038/s41467-023-40219-8)
Supplement: Supplementary file 1 — Supplementary Information [file 41467_2023_40219_MOESM1_ESM.pdf]

Supplementary Information

Macrocyclization of linear molecules by deep learning to facilitate macrocyclic drug candidates discovery

Yanyan Diao[1], Dandan Liu[1], Huan Ge[1], Rongrong Zhang[1], Kexin Jiang[1], Runhui Bao[1], Xiaoqian Zhu[1], Hongjie Bi[1], Wenjie Liao[1], Ziqi Chen[1], Kai Zhang[2], Rui Wang[1], Lili Zhu[1], Zhenjiang Zhao[1], Qiaoyu Hu[2], Honglin Li[1,2,3,*]

[1] Shanghai Key Laboratory of New Drug Design, School of Pharmacy, East China University of Science & Technology, Shanghai 200237, China

[2] Innovation Center for AI and Drug Discovery, East China Normal University, Shanghai 200062, China

[3] Lingang Laboratory, Shanghai 200031, China

*To whom correspondence should be addressed. Email: hlli@ecust.edu.cn

Contents of Supplementary Information

Supplementary Fig. 1. Training loss of four models with different augmentation levels. 3

Supplementary Fig. 2. The workflow of the traditional non-deep learning macrocylization method MacLS. 4

Supplementary Fig. 3. Distribution of molecular weight (a) and SMILES length (b) of collected macrocycles. 5

Supplementary Fig. 4. Distribution of molecular properties using ChEMBL test dataset. 6

Supplementary Fig. 5. Distribution of molecular properties using ZINC dataset. 8

Supplementary Fig. 6. Attention weights analysis. 10

Supplementary Fig. 7. Docking scores distribution of macrocyclic analogues of Fedratinib generated by Macformer and MacLS, respectively. 11

Supplementary Fig. 8. Plasma concentration vs time curves after iv (5 mg/kg) or po (5 mg/kg) administration of compounds **1** (a) and **3** (b) and Fedratinib (c), respectively. 12

Supplementary Methods...13

Supplementary Fig. 9. The procedure for the synthesis of compound **1**. 14

Supplementary Fig.10. The procedure for the synthesis of compound **2**. 17

Supplementary Fig. 11. The procedure for the synthesis of compound **3**. 21

Supplementary Fig. 12. NMR spectrometry data of compound **1**. a 13C NMR spectrometry data, b 1H NMR spectrometry data. 25

Supplementary Fig. 13. HRMS (ESI) spectrometry data of compound **1**. 26

Supplementary Fig. 14. NMR spectrometry data of compound **2**. 27

Supplementary Fig. 15. HRMS (ESI) spectrometry data of compound **2**. 28

Supplementary Fig. 16. NMR spectrometry data of compound **3**. 29

Supplementary Fig. 17. HRMS (ESI) spectrometry data of compound **3**. 30

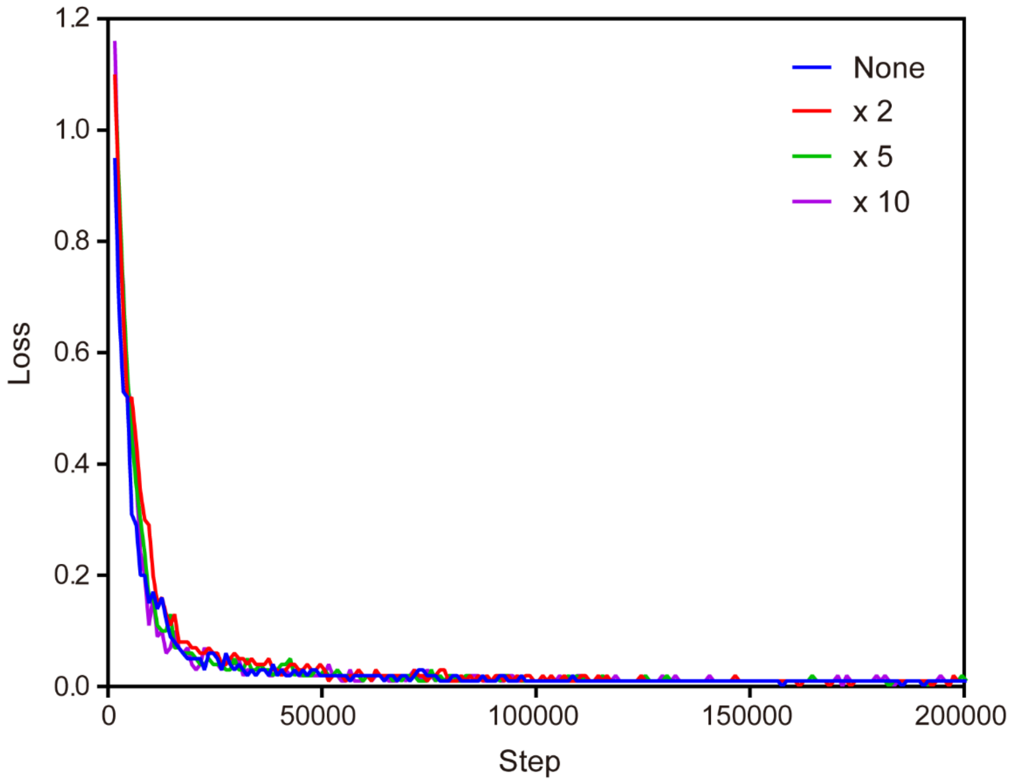

Supplementary Fig. 1. Training loss of four models with different augmentation levels.

For each model, n = 200 checkpoints. Source data are provided as a Source Data file.

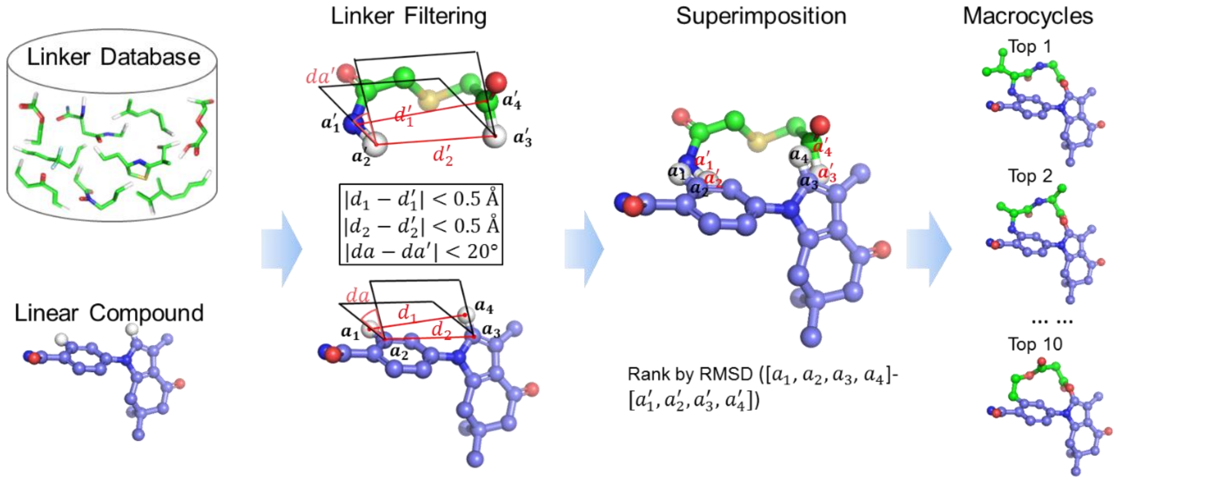

Supplementary Fig. 2. The workflow of the traditional non-deep learning macrocylization method MacLS. The white atoms present the dummy atoms that label the positions of the broken bonds (attachment vectors). When a macrocycle is formed, the leaving atoms a_1 and a_4 in Fedratinib and atoms a_2' and a_3' in the linker will not be contained in the macrocycle. Ideally, the differences between distances ($|d_1 - d_1'|$ and $|d_2 - d_2'|$) and dihedral angles ($|da - da'|$) should be 0. The parameter da is the dihedral angle between atoms a_1, a_2, a_3, and a_4 in Fedratinib, and $da'$is the dihedral angle between atoms a_1', a_2', a_3', and a_4' in the linker. The bigger difference values means the worse compatibility between Fedratinib and the linker. The bigger difference values means the worse compatibility between Fedratinib and the linker. The broken bonds of the linkers that satisfy the geometric criteria were superimposed to that of the linear compound, and the RMSD values were calculated to rank the linkers. Top 10 linkers were connected to the linear compound to generate macrocycles.

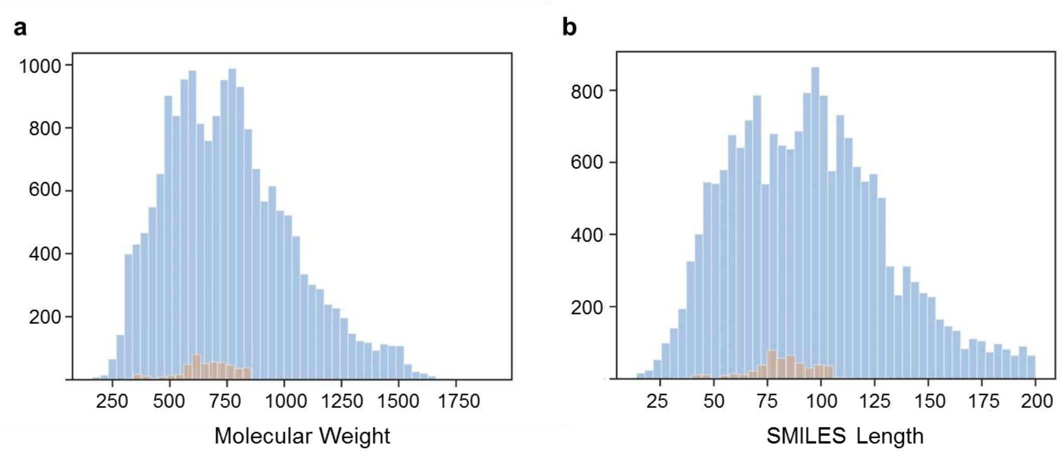

Supplementary Fig. 3. Distribution of molecular weight (a) and SMILES length (b) of collected macrocycles. ChEMBL, blue, $n = 18357$ molecules; ZINC, yellow, $n = 486$ molecules. Source data are provided as a Source Data file.

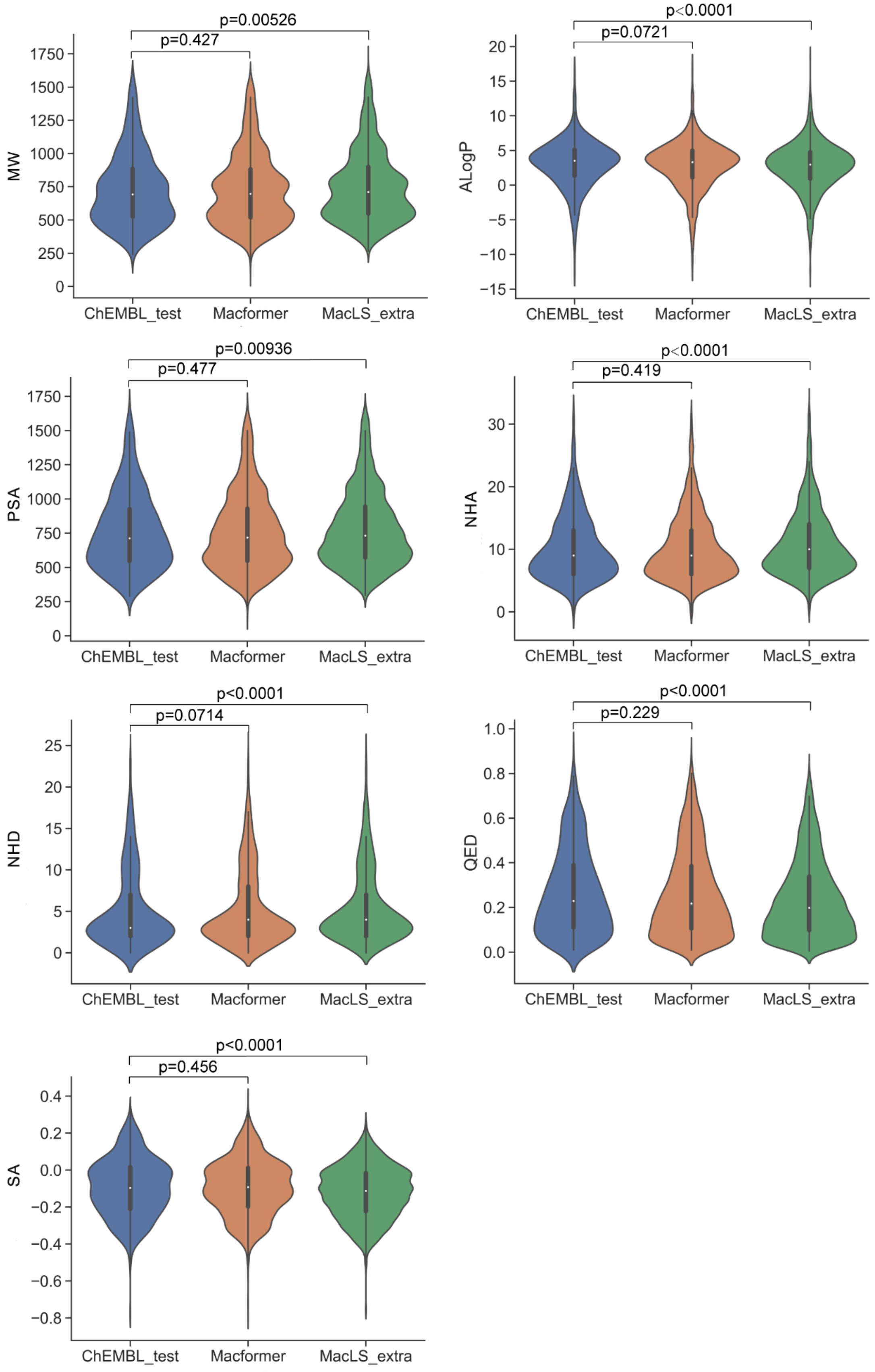

Supplementary Fig. 4. Distribution of molecular properties using ChEMBL test dataset.

Target macrocycles in ChEMBL test dataset, *n* = 939 molecules; generated novel macrocycles

by Macformer with 5-fold augmentation, n = 5717 molecules; generated novel macrocycles by MacLS_extra, n = 9552 molecules. We randomly selected 1000 target macrocycles and their corresponding generated novel compounds for clarity, and the duplicate molecules were removed. The properties include molecular weight (MW), AlogP, polar surface area (PSA), the number of hydrogen bond acceptors (NHA), the number of hydrogen bond donors (NHD), quantitative estimates of drug-likeness (QED), and synthetic accessibility (SA). The white dot shows the median value and the black box shows the interquartile range. Two-sided Wilcoxon rank-sum test was used to calculate the statistical difference of each distribution with respect to the target macrocycles. Source data are provided as a Source Data file.

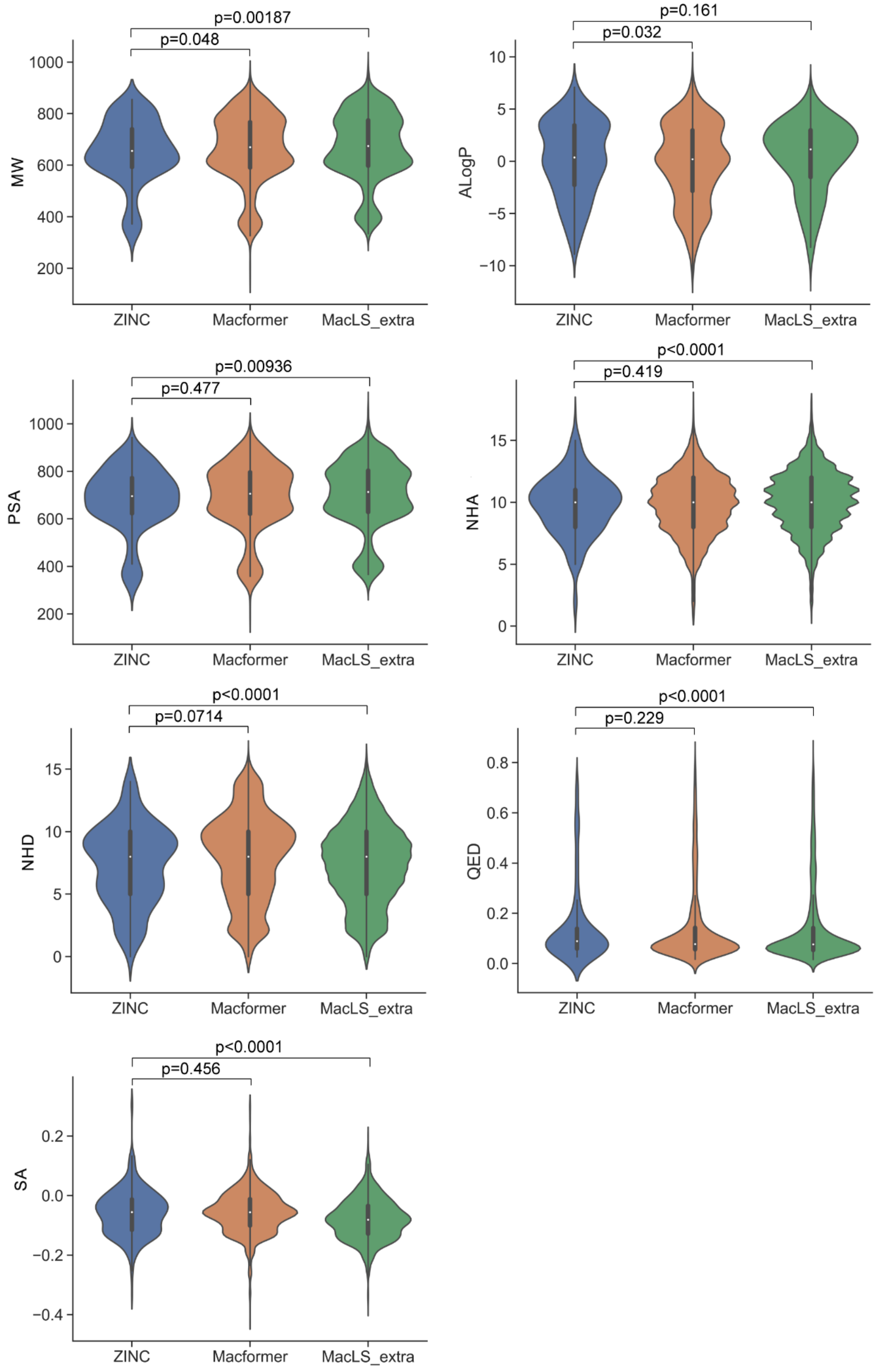

Supplementary Fig. 5. Distribution of molecular properties using ZINC dataset. Target macrocycles in ZINC dataset, *n* = 354 molecules; generated novel macrocycles by Macformer

with 5-fold augmentation, n = 4863 molecules; generated novel macrocycles by MacLS_extra, n = 9477 molecules. We randomly selected 1000 target macrocycles and their corresponding generated novel compounds for clarity, and the duplicate molecules were removed. The properties include molecular weight (MW), AlogP, polar surface area (PSA), the number of hydrogen bond acceptors (NHA), the number of hydrogen bond donors (NHD), quantitative estimates of drug-likeness (QED), and synthetic accessibility (SA). The white dot shows the median value and the black box shows the interquartile range. Two-sided Wilcoxon rank-sum test was used to calculate the statistical difference of each distribution with respect to the target macrocycles. Source data are provided as a Source Data file.

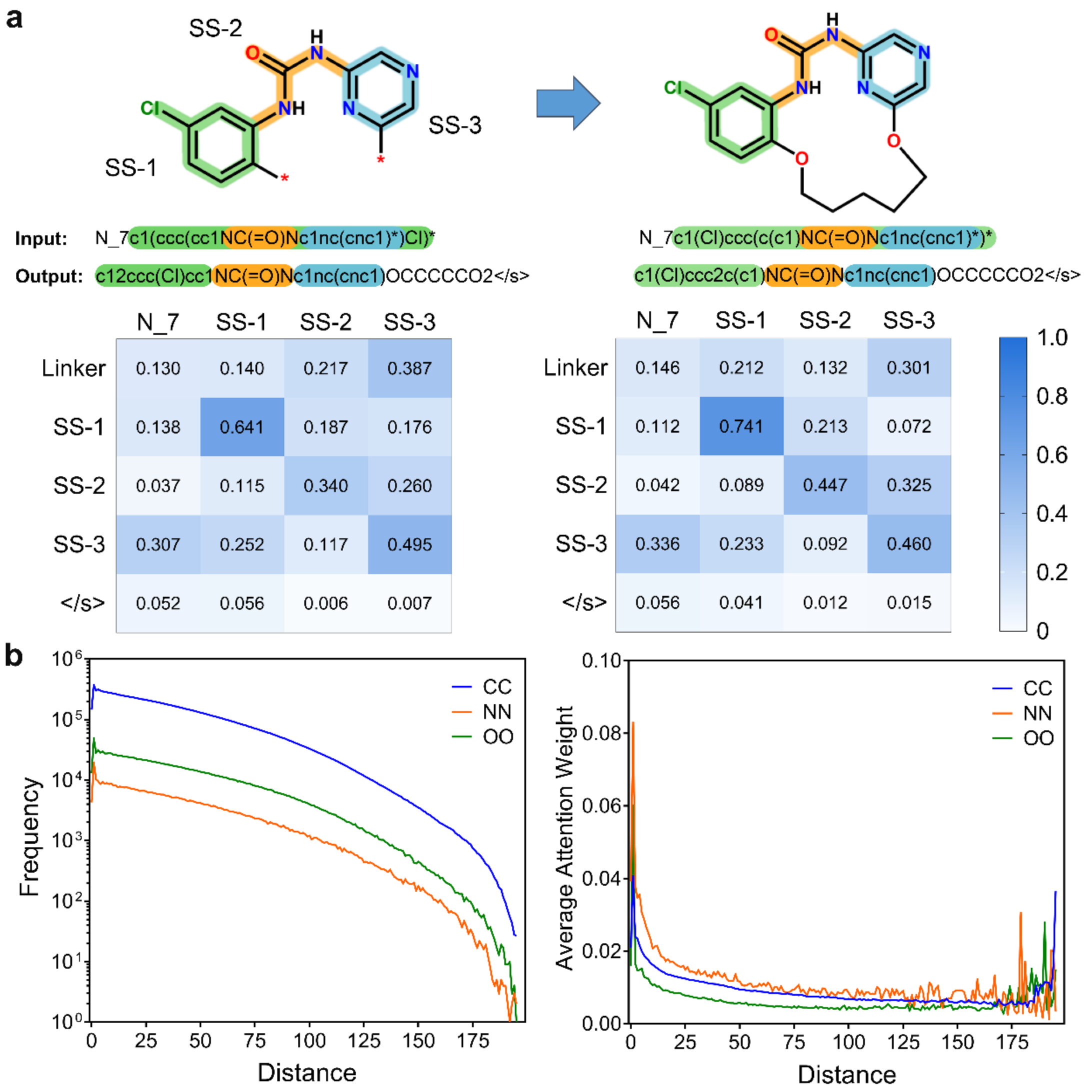

Supplementary Fig. 6. Attention weights analysis. a Heatmap representation of average attention weights between substrings from input and output sequences, respectively. To simplify the representation of attention matrix produced by long sequences, the SMILES strings were divided into several sections according to the substructures, and the average attention weights were calculated. **b** Distributions of frequencies and average attention weights of CC, NN, and OO pairs at different distances. The distance was defined as the relative value of positions in the input and output sequences. These data were extracted from ChEMBL test dataset with confidence scores, the product of the probabilities of all predicted tokens, more than 0.8. The higher confidence score implies the more confidence of the model's prediction. Source data are provided as a Source Data file.

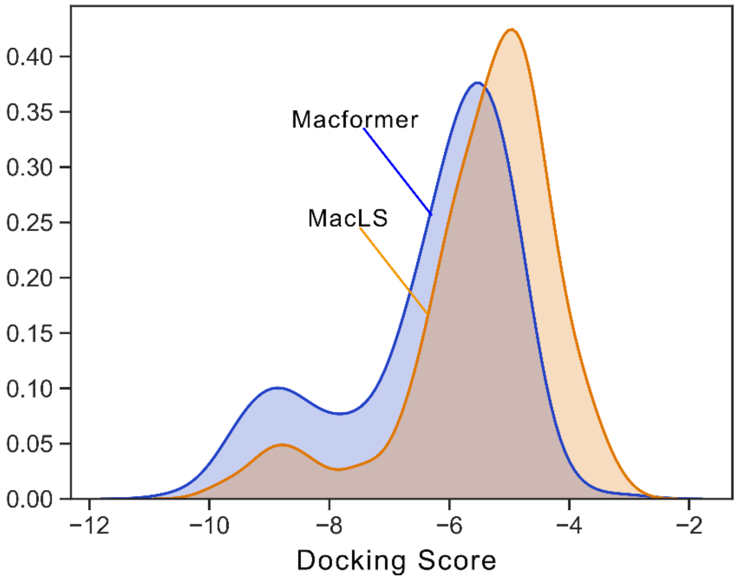

Supplementary Fig. 7. Docking scores distribution of macrocyclic analogues of Fedratinib generated by Macformer and MacLS, respectively. Source data are provided as a Source Data file.

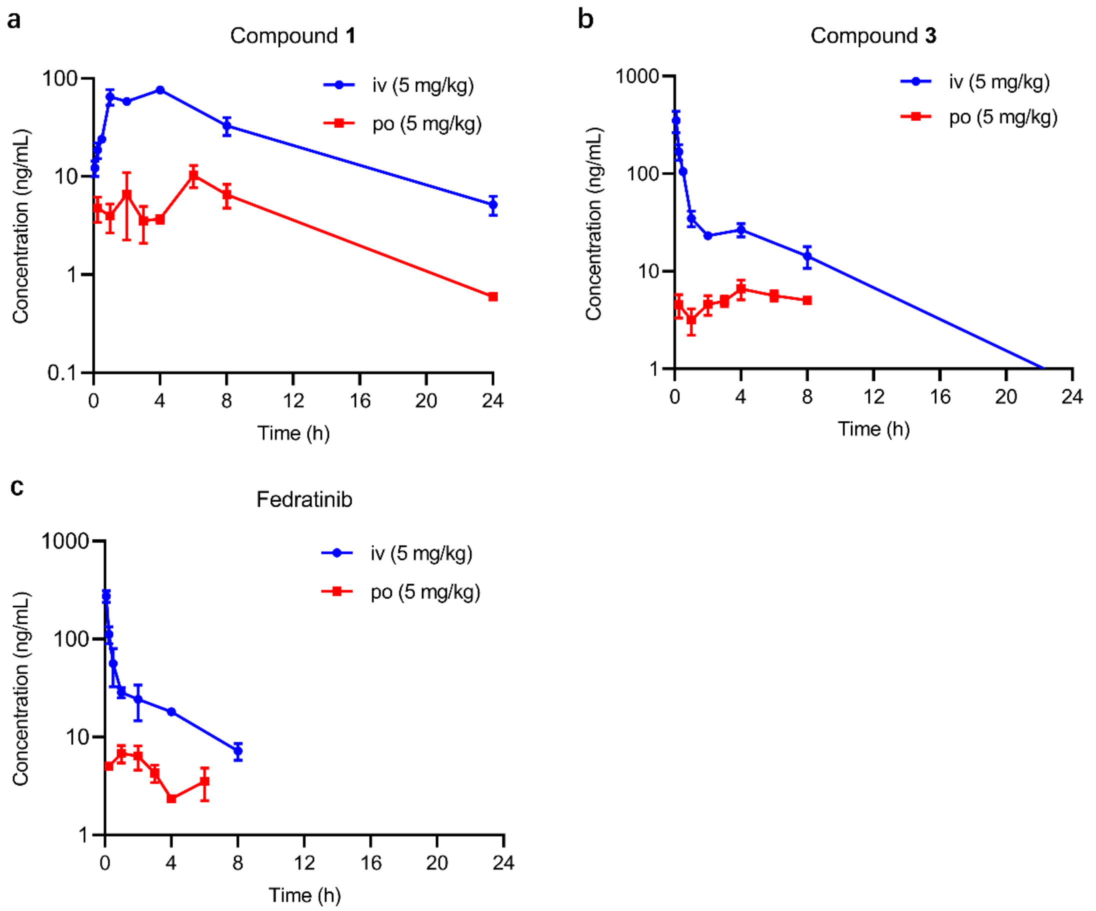

Supplementary Fig. 8. Plasma concentration vs time curves after iv (5 mg/kg) or po (5 mg/kg) administration of compounds 1 (a) and 3 (b) and Fedratinib (c), respectively. Data are represented as the mean ± SD (n = 3 mice). Source data are provided as a Source Data file.

Supplementary Methods

The reagents used in chemical experiments were all commercial reagents, and the solvents used were analytically pure (AR) grades and were used directly without purification, most of which were purchased from Energy Chemical Company. All the reactions were monitored by thin layer chromatography (TLC) on F-254 silica gel plate and observed under UV light. Mass spectra for all intermediates were recorded on an Agilent Technologies 6100 Series Single Quadrupole LC/MS or Micromass GCT CA 055 instrument. ^{1}H NMR and ^{13}C NMR spectra were performed using Brucker Avance-400 (400 MHz) or Ascend 600 (600 MHz) spetrometer in $CDCl_3$ or DMSO-d_6 with TMS as an internal standard. High-resolution mass spectra (HRMS) were acquired with a Xevo G2 TOF MS spectrometer in positive ESI mode. Melting points (m.p.) were taken on a WRS-1B digital melting point apparatus and uncorrected. The purity of all final compounds were performed on an Agilent 1100 series HPLC using an Agilent XDB 5 μ C18 column (4.6 mm×150 mm), elution with a mixture of 20% H_2O and 80% Methanol, the flow rate of the mobile phase was 0.8 mL/min and the injection volume was 10 μL, in addition, the determined wavelength was 254 nm.

Synthesis of compound 1.

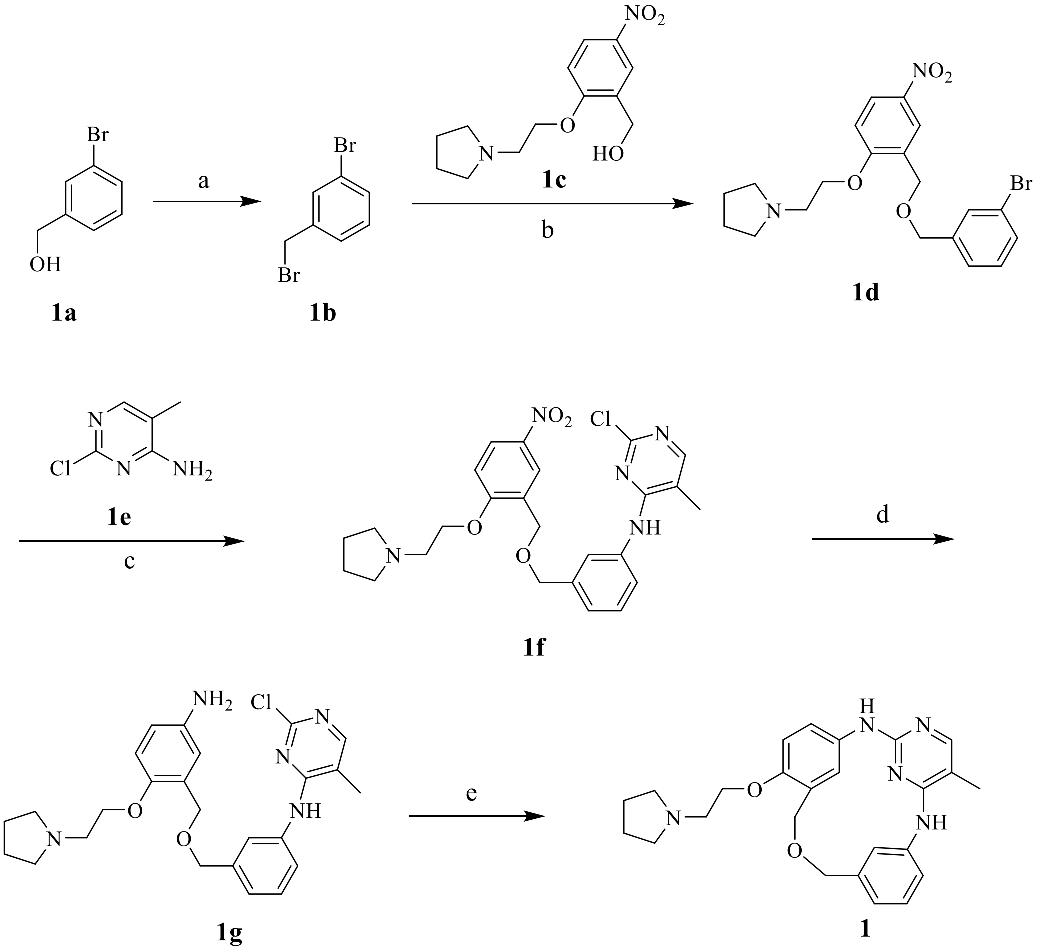

Supplementary Fig. 9. The procedure for the synthesis of compound 1. Reagents and conditions: (a) PBr_3, DCM, 0 ℃, 1 h; (b) NaH, DMF, -20 ℃ -r.t., 1.5 h, 52.0%; (c) CS_2CO_3, Xantphos, $Pd_2(dba)_3$, DMF, 80 ℃, 8 h, 28.5%; (d) $N_2H_4 \cdot H_2O$, $FeCl_3$, EtOH, 80 ℃, 1 h, 39.0%; (e) TsOH, n-butanol, 105 ℃, 2 h, 35.5%.

1-bromo-3-(bromomethyl)benzene (1b). Phosphorus tribromide (1 mL) was added to a solution of 3-bromobenzyl alcohol (**1a)** (5.00 g, 26.73 mmol) in dichloromethane (30 mL) at 0 °C. The reaction solution was stirred under an ice bath for 1 h. Then, the reaction solution was extracted with DCM, dried with anhydrous Na_2SO_4, and concentrated in vacuum to give the product as a colorless oil, the product can be directly used in the next step.

1-(2-(2-(((3-bromobenzyl)oxy)methyl)-4-nitrophenoxy)ethyl)pyrrolidine (1d). Sodium Hydride (225 mg, 5.63 mmol) was added into (5-nitro-2-(2-(pyrrolidin-1-yl)ethoxy)phenyl)methanol (**1c)** (1.20 g, 4.51 mmol) and then rapidly replaced with N_2 for 3 times. DMF (10 mL) was added and stirred for 30 min at -20 °C . Then, **1b** (1.46 g, 5.86 mmol)

was slowly administered through a constant pressure funnel. The reaction solution was stirred for 1.5 h at room temperature. Then, the reaction solution was quenched slowly with H_2O, extracted with EA, dried with anhydrous Na_2SO_4, and concentrated with rotary evaporator. The crude product was purified by silica gel chromatography (DCM: MeOH = 50: 1, v/v) to obtain the title product yellow oil (1.02 g, 52.0%).^{1}H NMR (400 MHz, DMSO-d_6) δ 8.20 – 8.14 (m, 2H), 7.53 (m, 1H), 7.46 (m, 1H), 7.32 (m, 2H), 7.18 (d, *J* = 8.7 Hz, 1H), 4.60 (s, 2H), 4.53 (s, 2H), 4.20 (t, *J* = 5.6 Hz, 2H), 2.78 (t, *J* = 5.6 Hz, 2H), 2.46 (m, 4H), 1.61 (m, 4H). LC-MS: m/z: 435.10 $[M+H]^+$.

2-chloro-5-methyl-N-(3-(((5-nitro-2-(2-(pyrrolidin-1-yl)ethoxy)benzyl)oxy)-methyl)phenyl)pyrimidin-4-amine (1f) 2-chloro-5-methylpyrimidin-4-amine **(1e).** (0.41 g, 2.81 mmol), Cs_2CO_3(2.29 g, 7.02 mmol) , Xantphos (0.27 g, 0.47 mmol) , $Pd_2(dba)_3$ (0.63 g, 0.70 mmol) were added to **1d** (1.02 g, 2.34 mmol) and then rapidly replaced with N_2 for 3 times. DMF (15 mL) was added. The reaction solution was stirred for 8 h at 80 ℃. Then, the reaction liquid was quenched slowly by adding H_2O, extracted with EA, washed with saturated aqueous NaCl, dried with anhydrous Na_2SO_4, and concentrated at reduced pressure. The crude product was purified by silica gel chromatography (DCM: MeOH = 40: 1, v/v) to obtain the title product yellow oil (0.33 g, 28.5%).^{1}H NMR (400 MHz, DMSO-d_6) δ 8.92 (s, 1H), 8.22 (s, 1H), 8.18 (m, 1H), 8.02 (s, 1H), 7.65 (s, 1H), 7.60 (d, *J* = 7.5 Hz, 1H), 7.35 (t, *J* = 7.8 Hz, 1H), 7.21 (d, *J* = 9.1 Hz, 1H), 7.11 (d, *J* = 7.5 Hz, 1H), 4.63 (s, 2H), 4.57 (s, 2H), 4.21 (t, *J* = 5.5 Hz, 2H), 2.78 (t, *J* = 5.5 Hz, 2H), 2.45 (m, 4H), 2.15 (s, 3H), 1.60 (m, 4H). LC-MS: m/z: 498.20 $[M+H]^+$.

N-(3-(((5-amino-2-(2-(pyrrolidin-1-yl)ethoxy)benzyl)oxy)methyl)phenyl)-2-chloro-5-methylpyrimidin-4-amine (1g). Anhydrous $FeCl_3$ (0.13 g, 2.65 mmol), hydrazine hydrate (11 mg, 0.07 mmol) and activated carbon was added to a solution of **1f** (5.00 g, 26.73 mmol) in anhydrous ethanol (30 mL).The reaction solution was stirred at 80 °C for 1 h. Then, Reactive liquid was filtered to remove activated carbon, extracted with EA, dried with anhydrous Na_2SO_4, and concentrated in vacuo to give the product as a white solid. The product can be used directly in the next step.

3^5-methyl-1^4-(2-(pyrrolidin-1-yl)ethoxy)-7-oxa-2,4-diaza-3(2,4)-pyrimidina-1,5(1,3)-dibenzenacyclooctaphane (1). P-toluene sulfonic acid (88 mg, 0.52 mmol) was added to a

solution of **1g** (5.00 g, 26.73 mmol) in n-butanol (80 mL). The reaction solution was stirred at 105 °C for 2 h. Then, the reaction liquid was quenched slowly by adding H_2O, extracted with EA and the water phase was adjusted to PH > 8 with saturated sodium bicarbonate solution. The organic phase was dried with anhydrous Na_2SO_4 and distilled under reduced pressure. The crude product was purified by silica gel chromatography (DCM: MeOH = 7: 1, v/v) to obtain the title product yellow oil (39 mg, 35.5%).^{1}H NMR (600 MHz, Chloroform-*d*) δ 8.58 (s, 1H), 8.37 (s, 1H), 7.86 (s, 1H), 7.12 (s, 1H), 7.05 (d, *J* = 7.7 Hz, 1H), 6.86 (dd, *J* = 7.9, 1.6 Hz, 1H), 6.78 (m, 2H), 6.38 (s, 1H), 4.65 (s, 2H), 4.56 (s, 2H), 4.10 (t, *J* = 6.0 Hz, 2H), 2.92 (t, *J* = 6.0 Hz, 2H), 2.65 (m, 4H), 2.11 (s, 3H), 1.83 (m, 4H).^{13}C NMR (151 MHz, Chloroform-*d*) δ 159.23, 159.05, 156.25, 152.49, 139.17, 138.90, 133.33, 128.60, 126.83, 124.79, 124.76, 123.31, 119.83, 119.73, 112.12, 104.57, 71.51, 68.17, 66.09, 55.21, 54.87, 23.64, 13.38. HRMS (ESI): (m/z): $[M+H]^+$ calcd for $C_{25}H_{29}N_5O_2$, 432.2321; found: 432.2401. HPLC purity: 94.66%, retention time = 1.460 min.

Synthesis of compound 2.

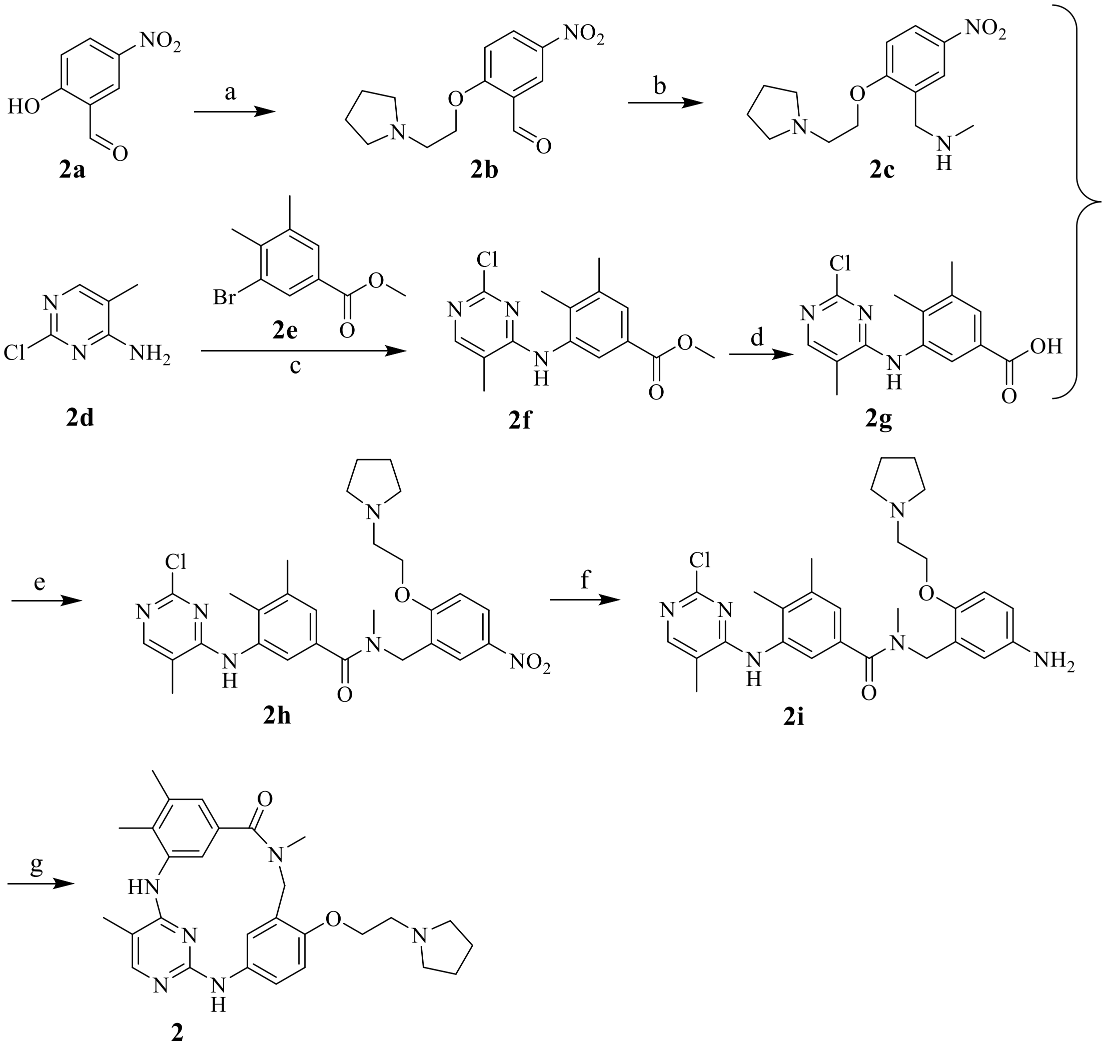

Supplementary Fig. 10. The procedure for the synthesis of compound 2. Reagents and conditions: (a) 1-(2-Chloroethyl)pyrrolidine Hydrochloride, Cs_2CO_3, DMF, 100 ℃, 12h, 58.3%; (b) $CH_3NH_2 \cdot HCl$, $NaBH_3CN$, MeOH, r.t., 12 h, 73.9%; (c) Cs_2CO_3, Xantphos, $Pd_2(dba)_3$, dioxane, reflux, 5 h, 46.5-51.3%; (d) NaOH, H_2O, MeOH, 40 ℃, 40 min, 73.9-77.9%; (e) HATU, DIPEA, DMF, r.t., 1 h, 50.3-55.3%; (f) 10% Pd/C, H_2, MeOH, 40 ℃, 40 min, 45.3-50.1%; (g) Cs_2CO_3, Xantphos, $Pd_2(dba)_3$, DMF, 140 ℃, 6 h, 50.4-55.9%.

5-nitro-2-(2-(pyrrolidin-1-yl)ethoxy)benzaldehyde (2b). 2-hydroxy-5-nitrobenzaldehyde (0.50 g, 2.99 mmol), 1-(2-chloroethyl)pyrrolidine Hydrochloride (0.71 g, 4.19 mmol), Cs_2CO_3 (1.95 g, 5.98 mmol) were added to the reaction flask, anhydrous DMF (8 mL) was added in a N_2 atmosphere. The mixture was stirred at 100°C for about 12 h, then cooled and extracted with CH_2Cl_2, combined the organic phases, dried over anhydrous Na_2SO_4, and concentrated under

reduced pressure. The mixture was separated by column chromatography using CH_2Cl_2/MeOH (30: 1) as eluent to afford 0.45 g of **2b** as a yellow solid; yield: 58.3%. ^{1}H NMR (400 MHz, DMSO-*d*$_6$) δ 10.34 (s, 1H), 8.49 (dd, *J* = 9.2, 2.9 Hz, 1H), 8.41 (d, *J* = 2.9 Hz, 1H), 7.50 (d, *J* = 9.2 Hz, 1H), 4.41 (t, *J* = 5.4 Hz, 2H), 2.95 (t, *J* = 5.2 Hz, 2H), 2.64-2.58 (m, 4H), 1.72-1.68 (m, 4H). LC-MS (ESI): m/z: 265.20 $[M+H]^+$.

N-methyl-1-(5-nitro-2-(2-(pyrrolidin-1-yl)ethoxy)phenyl)methanamine (2c). The intermediate **2b** (0.32 g, 1.21 mmol), CH_3NH_2·HCl (0.16 g, 2.42 mmol), $NaBH_3CN$ (91 mg, 1.45 mmol) were dissolved in MeOH (5 mL), then stirred at room temperature for about 12 h in a N_2 atmosphere. After the mixture was extracted with EtOAc, combined the organic phases and washed with brine, dried over anhydrous Na_2SO_4, and concentrated under reduced pressure. The mixture was separated by column chromatography using CH_2Cl_2/MeOH (10: 1) as eluent to afford 0.25 g of **2c** as a yellow solid; yield: 73.9%. ^{1}H NMR (400 MHz, DMSO-*d*$_6$) δ 8.18 (s, 1H), 8.11 (d, *J* = 8.9 Hz, 1H), 7.18 (d, *J* = 9.0 Hz, 1H), 4.21 (t, *J* = 5.5 Hz, 2H), 3.63 (s, 2H), 2.81 (t, *J* = 5.5 Hz, 2H), 2.50-2.41 (m, 4H), 2.25 (s, 3H), 1.69-1.62 (m, 4H). LC-MS (ESI): m/z: 280.20 $[M+H]^+$.

Methyl 3-((2-chloro-5-methylpyrimidin-4-yl)amino)benzoate (2f). 2-chloro-5-methylpyrimidin-4-amine (0.50 g, 3.48 mmol), methyl 3-bromobenzoate (0.90 g, 4.18 mmol), Cs_2CO_3 (3.40 g, 10.45 mmol), Xantphos (0.40 g, 0. 70 mmol) and $Pd_2(dba)_3$ (0.32 g, 0.35 mmol) were placed in a reaction flask, anhydrous 1,4-dioxane (8 mL) was added in a N_2 atmosphere. The mixture was stirred under reflux for about 5 h, then filtered and extracted with CH_2Cl_2. The organic phases were combined, dried over anhydrous Na_2SO_4. The mixture was purified by column chromatography using PE/EtOAc (5: 1) as eluent to afford 0.45 g of **2f** as a yellow solid; yield: 46.5%. ^{1}H NMR (400 MHz, DMSO-*d*$_6$) δ 9.05 (s, 1H), 8.26 (s, 1H), 8.09 (s, 1H), 8.03 (d, *J* = 8.1, 1.2 Hz, 1H), 7.69 (d, *J* = 7.8 Hz, 1H), 7.52 (t, *J* = 7.9 Hz, 1H), 3.87 (s, 3H), 2.19 (s, 3H). LC-MS (ESI): m/z: 278.10/280.10 $[M+H]^+$.

3-((2-chloro-5-methylpyrimidin-4-yl)amino)benzoic acid (2g). The intermediate **2f** (0.10 g, 0.36 mmol) was dissolved in MeOH (30 mL), added saturated aqueous NaOH (0.35 g, 8.64 mmol). The mixture was stirred at 40°C for about 40 min, then the MeOH was removed and the pH was adjusted to 2 with 1M HCl, extracted with EtOAc, the organic phases were combined and dried over anhydrous Na_2SO_4, and concentrated under reduced pressure to obtain

0.74 g of **2g** as a brown solid; yield: 77.9%. ^{1}H NMR (400 MHz, CD_3OD) δ 8.24 (s, 1H), 7.99-7.92 (m, 2H), 7.80 (d, J = 7.7 Hz, 1H), 7.46 (t, J = 7.9 Hz, 1H), 2.21 (s, 3H). LC-MS (ESI): m/z: 264.10/266.10 $[M+H]^+$.

3-((2-chloro-5-methylpyrimidin-4-yl)amino)-N-methyl-N-(5-nitro-2-(2-(pyrrolidin-1-yl) ethoxy)benzyl)benzamide (2h). The intermediate **2g** (34 mg, 0.13 mmol), intermediate **3** (43.17 mg, 0.15 mmol) and DIPEA (166.60 mg, 1.29 mmol) were dissolved in DMF (2 mL), added HATU (68.60 mg, 0.18 mmol), then stirred at room temperature for about 1 h. After the mixture was extracted with EtOAc, the organic phases were combined and washed with saturated aqueous $NaHCO_3$ and brine. The mixture was purified by column chromatography using CH_2Cl_2/MeOH (30: 1) as eluent to afford 36.55 mg of **2h** as a yellow solid; yield: 54.0%. ^{1}H NMR (400 MHz, CD_3OD) δ 7.88 (s, 1H), 7.82 (s, 1H), 7.76-7.67 (m, 2H), 7.48 (d, J = 7.9 Hz, 1H), 7.40 (t, J = 7.8 Hz, 1H), 7.28 (d, J = 7.4 Hz, 1H), 7.24 (d, J = 7.7 Hz, 1H), 6.84 (d, J = 8.5 Hz, 1H), 4.62 (s, 2H), 3.97 (t, J = 5.5 Hz, 2H), 3.03 (s, 3H), 2.74 (t, J = 5.5 Hz, 2H), 2.54 -2.46 (m, 4H), 2.03 (s, 3H), 1.76-1.69 (m, 4H). LC-MS (ESI): m/z: 525.20/527.20 $[M+H]^+$.

N-(5-amino-2-(2-(pyrrolidin-1-yl)ethoxy)benzyl)-3-((2-chloro-5-methylpyrimidin-4-yl) amino)-N-methylbenzamide (2i). The intermediate **2h** (0.68 g, 1.30 mmol) was dissolved in MeOH (10 mL), added 10% Pd/C (0.14 g, 20%), then stirred at 40 ℃ for about 40 min in a H_2 atmosphere. The mixture was filtered and concentrated under reduced pressure, purified by column chromatography using CH_2Cl_2/MeOH (30: 1) as eluent to afford 0.31 g of **2i** as a brown solid; yield: 48.4%. ^{1}H NMR (400 MHz, CD_3OD) δ 7.88 (s, 1H), 7.82 (s, 1H), 7.76-7.67 (m, 2H), 7.48 (d, J = 7.9 Hz, 1H), 7.40 (t, J = 7.8 Hz, 1H), 7.28 (d, J = 7.4 Hz, 1H), 7.24 (d, J = 7.7 Hz, 1H), 6.84 (d, J = 8.5 Hz, 1H), 4.76 (s, 2H), 4.62 (s, 2H), 3.97 (t, J = 5.5 Hz, 2H), 3.03 (s, 3H), 2.74 (t, J = 5.5 Hz, 2H), 2.54-2.46 (m, 4H), 2.03 (s, 3H), 1.76-1.69 (m, 4H). LC-MS (ESI): m/z: 495.20/497.20 $[M+H]^+$.

3^5,7-dimethyl-1^4-(2-(pyrrolidin-1-yl)ethoxy)-2,4,7-triaza-3(2,4)-pyrimidina-1,5(1,3)-dibenz enacyclooctaphan-6-one (2). The intermediate **2i** (50.00 mg, 0.10 mmol), Cs_2CO_3 (131.60 mg, 0.40 mmol), Xantphos (16.36 mg, 0. 03 mmol) and $Pd_2(dba)_3$ (18.50 mg, 0.02 mmol) were placed in a reaction flask, anhydrous DMF (1.5 mL) was added in a N_2 atmosphere. The mixture was stirred at 140°C for about 6 h, then filtered and extracted with CH_2Cl_2. The organic phases were combined, dried over anhydrous Na_2SO_4. The mixture was purified by

column chromatography using CH_2Cl_2/MeOH (20: 1) as eluent to afford 25.90 mg of **2** as a light yellow solid; yield: 55.9%. mp: >300 ℃. ^{1}H NMR (400 MHz, DMSO-d_6) δ 9.11 (s, 1H), 8.83 (d, J = 2.3 Hz, 1H), 8.72 (s, 1H), 7.89 (s, 1H), 7.65 (s, 1H), 7.42 (t, J = 7.8 Hz, 1H), 7.25 (d, J = 7.6 Hz, 1H), 7.06 (d, J = 7.3 Hz, 1H), 7.04-6.97 (m, 1H), 6.90 (d, J = 8.9 Hz, 1H), 4.27 (s, 2H), 4.03 (t, J = 5.6 Hz, 2H), 2.94 (s, 3H), 2.81 (t, J = 6.8 Hz, 2H), 2.57-2.52 (m, 4H), 2.11 (s, 3H), 1.74-1.65 (m, 4H). ^{13}C NMR (151 MHz, DMSO-d_6) δ 170.58, 159.24, 157.89, 156.77, 150.08, 139.64, 137.56, 135.26, 128.83, 124.70, 124.48, 122.75, 120.15, 119.67, 118.25, 112.92, 104.37, 67.48, 54.50, 54.11, 47.74, 32.20, 23.27, 13.45. HRMS (ESI): (m/z): $[M+H]^+$ calcd for $C_{26}H_{31}N_6O_2$ 459.2508; found 459.2507. HPLC purity: 96.7%, retention time = 6.20 min.

Synthesis of compound 3.

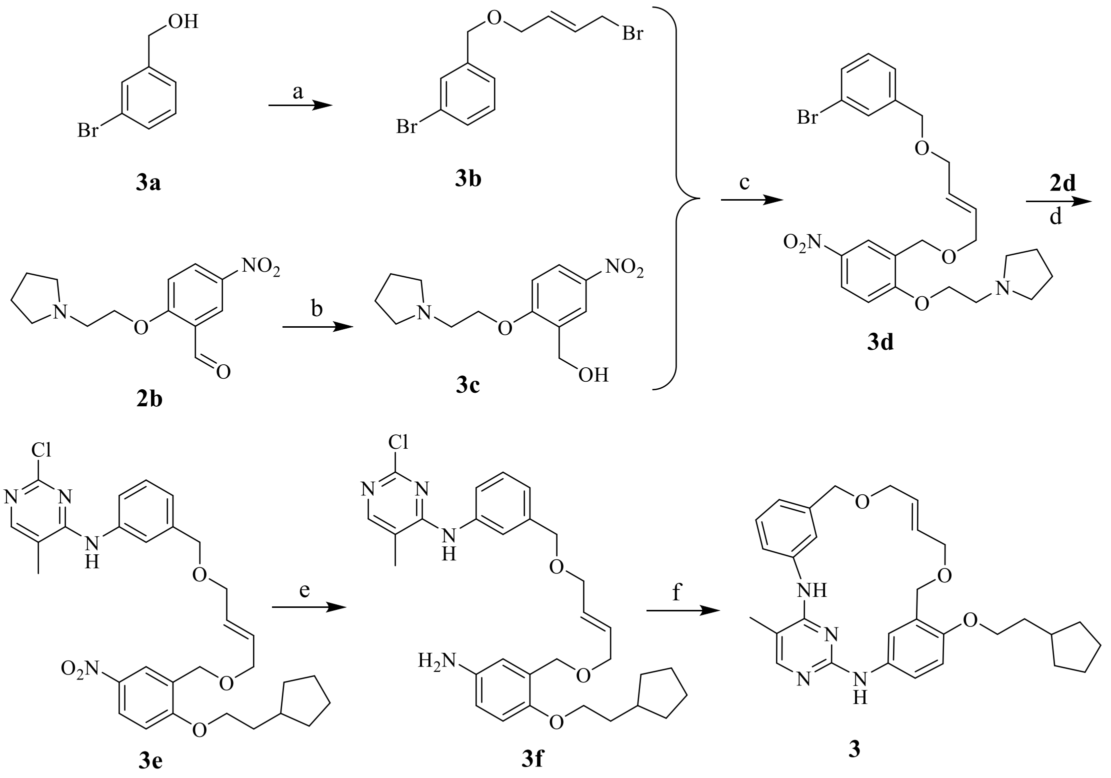

Supplementary Fig. 11. The procedure for the synthesis of compound 3. Reagents and conditions: (a) 1,4-dibromo-2-butene, NaOH, Bu_4NHSO_4, H_2O, CH_2Cl_2, r.t., 3 h, 40.9-48.3%; (b) $NaBH_4$, MeOH, r.t., 40 min, 59.5-63.3%; (c) NaH, DMF, 0 ℃, 1 h, 50.3-55.4%; (d) Cs_2CO_3, Xantphos, $Pd_2(dba)_3$, DMF, 80 ℃, 10 h, 65.3-72.2%; (e) $SnCl_2$, CH_2Cl_2, MeOH, r.t., 18 h, 37.2-42.3%; (f) TsOH·H_2O, n-butanol, 105 ℃, 2 h, 70.4-76.8%.

1-bromo-3-(((4-bromobut-2-en-1-yl)oxy)methyl)benzene (3b). (3-bromophenyl)methanol (0.20 g, 1.07 mmol) and 1,4-dibromo-2-butene (0.21 g, 0.96 mmol) were dissolved in CH_2Cl_2 (4 mL), slowly added saturated aqueous NaOH (0.34 g, 8.55 mmol), then stirred at room temperature for about 3 h. After the mixture was extracted with CH_2Cl_2, the organic phases were combined and concentrated under reduced pressure. The mixture was separated by column chromatography using PE/EtOAc (100: 1) as eluent to afford 0.14 g of **3b** as colorless oily liquid; yield: 40.9%. ^1H NMR (400 MHz, DMSO-d_6) δ 7.51 (s, 1H), 7.50-7.46 (m, 1H), 7.36-7.30 (m, 2H), 6.02-5.86 (m, 2H), 4.47 (s, 2H), 4.15 (dd, J = 4.5, 1.7 Hz, 2H), 4.02 (d, J = 3.1 Hz, 2H).

(5-nitro-2-(2-(pyrrolidin-1-yl)ethoxy)phenyl)methanol (3c). 5-nitro-2-(2-(pyrrolidin-1-yl)ethoxy)benzaldehyde (0.50 g, 1.89 mmol) was dissolved in MeOH (5 mL), slowly added

$NaBH_4$ (0.11 g, 2.84 mmol) under 0°C, and stirred at room temperature for about 40 min. After the methanol was removed, extracted with CH_2Cl_2, the organic phases were combined and dried over anhydrous Na_2SO_4. The mixture was separated by column chromatography using CH_2Cl_2/MeOH (40: 1) as eluent to afford 0.30 g of **3c** as a yellow solid; yield: 59.5%. ^{1}H NMR (400 MHz, DMSO-*d*$_6$) δ 8.24 (d, *J* = 2.8 Hz, 1H), 8.15 (dd, *J* = 9.0, 2.9 Hz, 1H), 7.18 (d, *J* = 9.1 Hz, 1H), 4.53 (s, 2H), 4.24 (t, *J* = 5.6 Hz, 2H), 2.85 (t, *J* = 5.6 Hz, 2H), 2.60-2.51 (m, 4H), 1.73-1.64 (m, 4H). LC-MS (ESI): m/z: 267.20 $[M+H]^+$.

1-(2-(2-(((4-((3-bromobenzyl)oxy)but-2-en-1-yl)oxy)methyl)-4-nitrophenoxy)ethyl)pyroli-dine (3d). The intermediate **3c** (100 mg, 0.38 mmol) and 60% NaH (18.78 mg, 0.47 mmol) were placed in a reaction flask. Under nitrogen protection, dry DMF (4 mL) was added at 0°C and stirred for 40 min, then **3b** (156.23 mg, 0.49 mmol) was added dropwise, and the mixture was stirred at room temperature for about 1 h. The reaction solution was poured into ice water, extracted with CH_2Cl_2, and the combined organic phase was washed with brine and dried with anhydrous Na_2SO_4. Purified by column chromatography using CH_2Cl_2/MeOH (50: 1) as eluent to obtain 100.58 mg of intermediate **3d** as a yellow solid; yield: 53.0%. ^{1}H NMR (400 MHz, DMSO-*d*$_6$) δ 8.18 (d, *J* = 7.3 Hz, 2H), 7.58-7.40 (m, 2H), 7.38-7.26 (m, 2H), 7.22 (d, *J* = 9.7 Hz, 1H), 5.87 (d, *J* = 18.6 Hz, 2H), 4.50 (s, 2H), 4.47 (s, 2H), 4.24 (t, *J* = 5.6 Hz, 2H), 4.10 (s, 2H), 4.02 (s, 2H), 2.81 (t, *J* = 5.6 Hz, 2H), 2.55-2.50 (m, 4H), 1.68-1.62 (m, 4H). LC-MS (ESI): m/z: 506.05 $[M+H]^+$.

2-chloro-5-methyl-N-(3-(((4-((5-nitro-2-(2-(pyrrolidin-1-yl)ethoxy)benzyl)oxy)but-2-en-1-yl)oxy)methyl)phenyl)pyrimidin-4-amine (3e). 2-chloro-5-methylpyrimidin-4-amine (0.12 g, 0.82 mmol), **3d** (0.50 g, 0.99 mmol), Cs_2CO_3 (0.97 g, 2.97 mmol), Xantphos (0.11 g, 0.20 mmol) and $Pd_2(dba)_3$ (0.09 g, 0.10 mmol) were placed in a reaction flask, anhydrous DMF (10 mL) was added in a N_2 atmosphere. The mixture was stirred at 80°C for about 10 h, then filtered and extracted with EtOAc. The organic phases were combined, dried over anhydrous Na_2SO_4. The mixture was purified by column chromatography using CH_2Cl_2/MeOH (50: 1) as eluent to afford 0.33 g of **3e** as a yellow solid; yield: 70.7%. ^{1}H NMR (400 MHz, DMSO-*d*$_6$) δ 8.90 (s, 1H), 8.18 (d, *J* = 7.4 Hz, 2H), 8.03 (s, 1H), 7.60 (s, 2H), 7.38 - 7.29 (m, 1H), 7.22 (d, *J* = 9.2 Hz, 2H), 7.06 (d, *J* = 7.2 Hz, 1H), 5.93-5.82 (m, 2H), 4.49 (d, *J* = 7.7 Hz, 4H), 4.23 (t, *J* = 5.4 Hz, 2H), 4.11 (s, 2H), 4.05 (s, 2H), 2.80 (t, *J* = 5.4 Hz, 2H), 2.55-2.50 (m, 4H), 2.16 (s,

3H), 1.68-1.62 (m, 4H).

2-chloro-N-(3-(((4-((2-methoxy-5-nitrobenzyl)oxy)but-2-en-1-yl)oxy)methyl)phenyl)-5-methylpyrimidin-4-amine (15d). Synthesized using the procedure for **15a** and 0.33 g of **15d** was obtained as a yellow solid; yield: 65.3%. ^{1}H NMR (400 MHz, DMSO-*d*$_6$) δ 8.90 (s, 1H), 8.21 (dd, *J* = 9.0, 2.8 Hz, 1H), 8.17 (d, *J* = 2.6 Hz, 1H), 8.03 (s, 1H), 7.61 (d, *J* = 6.7 Hz, 2H), 7.33 (t, *J* = 8.1 Hz, 1H), 7.21 (d, *J* = 9.0 Hz, 1H), 7.06 (d, *J* = 7.6 Hz, 1H), 5.88 (t, *J* = 2.6 Hz, 2H), 4.50 (d, *J* = 5.8 Hz, 4H), 4.12 (d, *J* = 1.4 Hz, 2H), 4.05 (d, *J* = 1.3 Hz, 2H), 3.92 (s, 3H), 2.16 (s, 3H).

N-(3-(((4-((5-amino-2-(2-(pyrrolidin-1-yl)ethoxy)benzyl)oxy)but-2-en-1-yl)oxy)methyl) phenyl)-2-chloro-5-methylpyrimidin-4-amine (3f). The intermediate **3e** (0.10 g, 0.18 mmol) was dissolved in mixed solution (4 mL) of CH_2Cl_2/MeOH (1: 1, v: v), and added $SnCl_2$ (0.13 g, 0.70 mmol) at 0°C. The mixture was stirred at room temperature for about 18 h. The solvent was removed, CH_2Cl_2 was added, the pH was adjusted to 8 with a saturated aqueous $NaHCO_3$, and then filtered and extracted with CH_2Cl_2. The organic phases were combined and dried over anhydrous Na_2SO_4, purified by column chromatography using CH_2Cl_2/MeOH (30: 1) as eluent to afford 39.50 mg of **3f** as a yellow solid; yield: 41.7%. ^{1}H NMR (400 MHz, DMSO-*d*$_6$) δ 8.91 (s, 1H), 8.04 (s, 1H), 7.62 (d, *J* = 8.2 Hz, 1H), 7.59 (s, 1H), 7.34 (t, *J* = 7.8 Hz, 1H), 7.06 (d, *J* = 7.5 Hz, 1H), 6.68 (d, *J* = 8.6 Hz, 1H), 6.60 (d, *J* = 2.5 Hz, 1H), 6.43 (dd, *J* = 8.5, 2.7 Hz, 1H), 5.88-5.79 (m, 2H), 4.63 (s, 2H), 4.48 (s, 2H), 4.36 (s, 2H), 4.03 (s, 2H), 4.00 (s, 2H), 3.90 (t, *J* = 5.9 Hz, 2H), 2.69 (t, *J* = 5.9 Hz, 2H), 2.49-2.42 (m, 4H), 2.17 (s, 3H), 1.67-1.59 (m, 4H). LC-MS (ESI): m/z: 539.25/541.20 $[M+H]^+$.

3^5-methyl-1^4-(2-(pyrrolidin-1-yl)ethoxy)-7,12-dioxa-2,4-diaza-3(2,4)-pyrimidina-1,5(1,3)-dibenzenacyclotridecaphan-9-ene (3). The intermediate **3f** (0.75 g, 1.39 mmol) was dissolved in n-butanol (15 mL), added TsOH·H_2O (0.53 g, 2.79 mmol) at 105 ℃. The mixture was stirred at 105 ℃ for about 2 h, then the pH was adjusted to 8-9 with a saturated aqueous Na_2CO_3, extracted with CH_2Cl_2. The organic phases were combined and and washed with brine, dried over anhydrous Na_2SO_4. The mixture was purified by column chromatography using CH_2Cl_2/MeOH (20: 1) as eluent to afford 0.52 g of **3** as a yellow solid; yield: 74.4%. mp: >300 ℃. ^{1}H NMR (400 MHz, DMSO-*d*$_6$) δ 8.64 (s, 1H), 8.30 (s, 1H), 7.85 (s, 1H), 7.61 (s, 1H), 7.55 (s, 1H), 7.28 (d, *J* = 7.7 Hz, 1H), 7.19-7.07 (m, 2H), 6.98 (d, *J* = 7.2 Hz, 1H), 6.79 (d,

J = 8.7 Hz, 1H), 5.56-5.36 (m, 2H), 4.30 (s, 2H), 4.14 (s, 2H), 4.02-3.93 (m, 4H), 3.84 (d, J = 3.7 Hz, 2H), 2.76 (t, J = 5.6 Hz, 2H), 2.58-2.51 (m, 4H), 2.08 (s, 3H), 1.72-1.64 (m, 4H). ^{13}C NMR (151 MHz, DMSO-d_6) δ 159.14, 158.78, 156.33, 151.42, 139.83, 139.75, 133.93, 130.02, 129.36, 127.93, 126.72, 122.25, 121.86, 121.55, 120.93, 112.17, 104.82, 71.66, 70.36, 69.32, 67.83, 66.67, 54.60, 54.29, 23.33, 13.57. HRMS (ESI): (m/z): $[M+H]^+$ calcd for $C_{29}H_{36}N_5O_3$ 502.2818; found 502.2820. HPLC purity: 98.7%, retention time = 5.91 min.

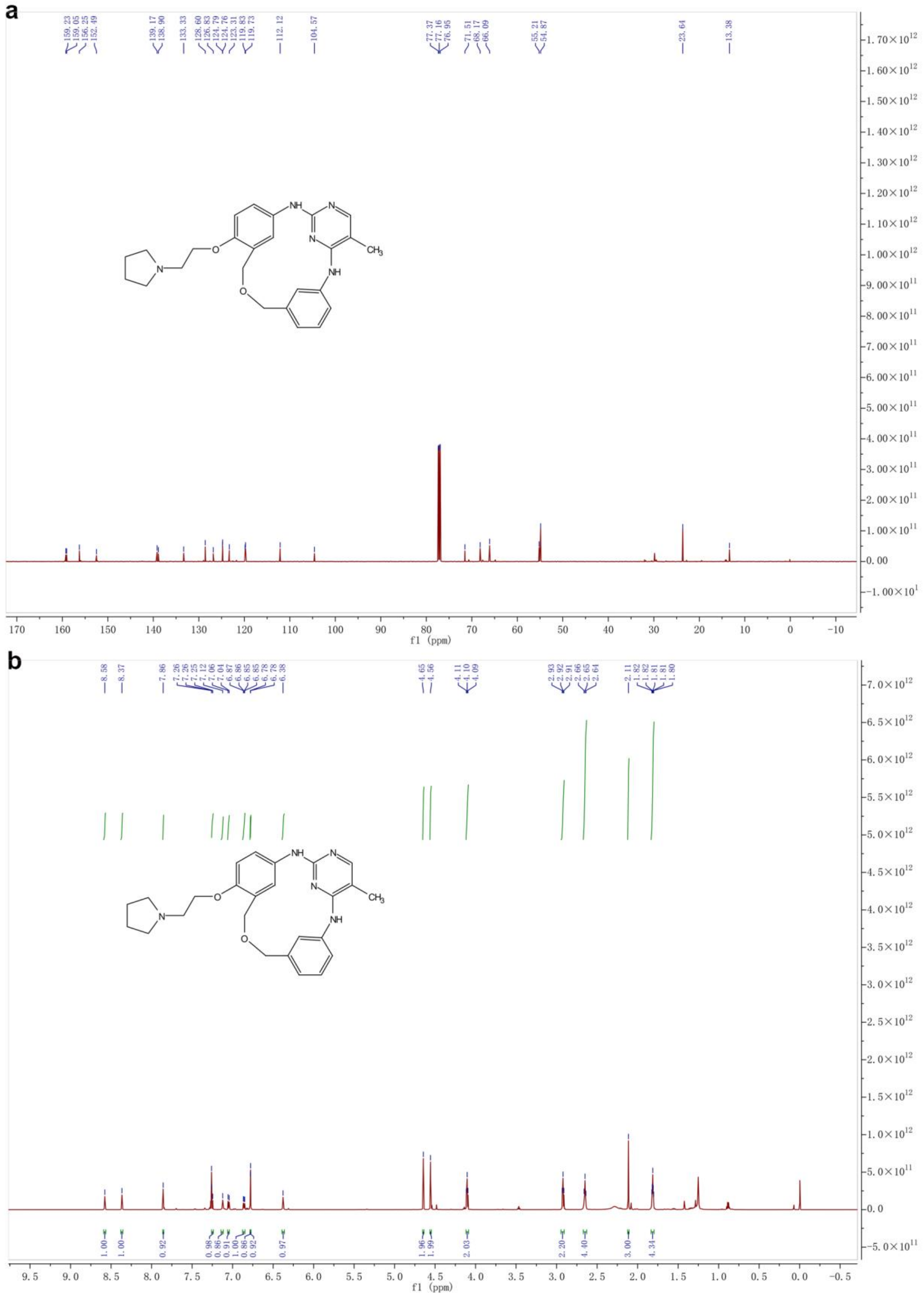

Supplementary Fig. 12. NMR spectrometry data of compound 1. a ^{13}C NMR spectrometry data, **b** ^{1}H NMR spectrometry data.

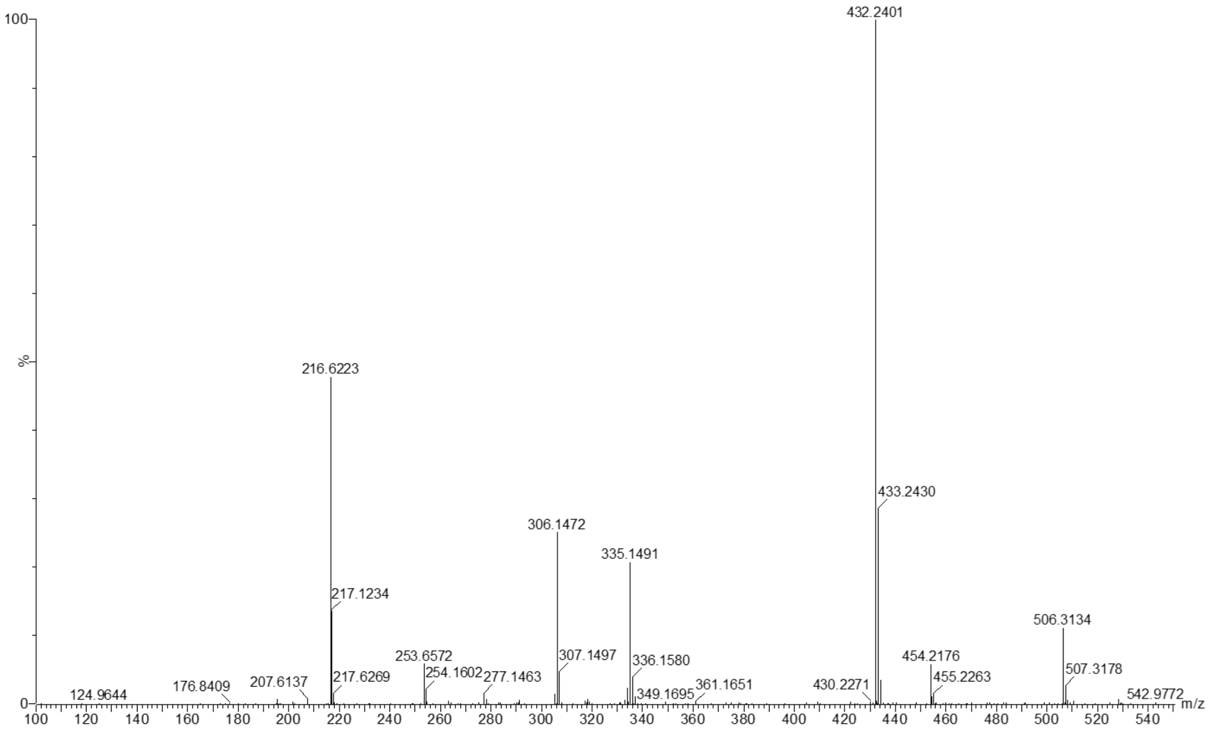

Supplementary Fig. 13. HRMS (ESI) spectrometry data of compound 1. $[M+H]^+$ calcd for $C_{25}H_{29}N_5O_2$, 432.2321; found: 432.2401.

a

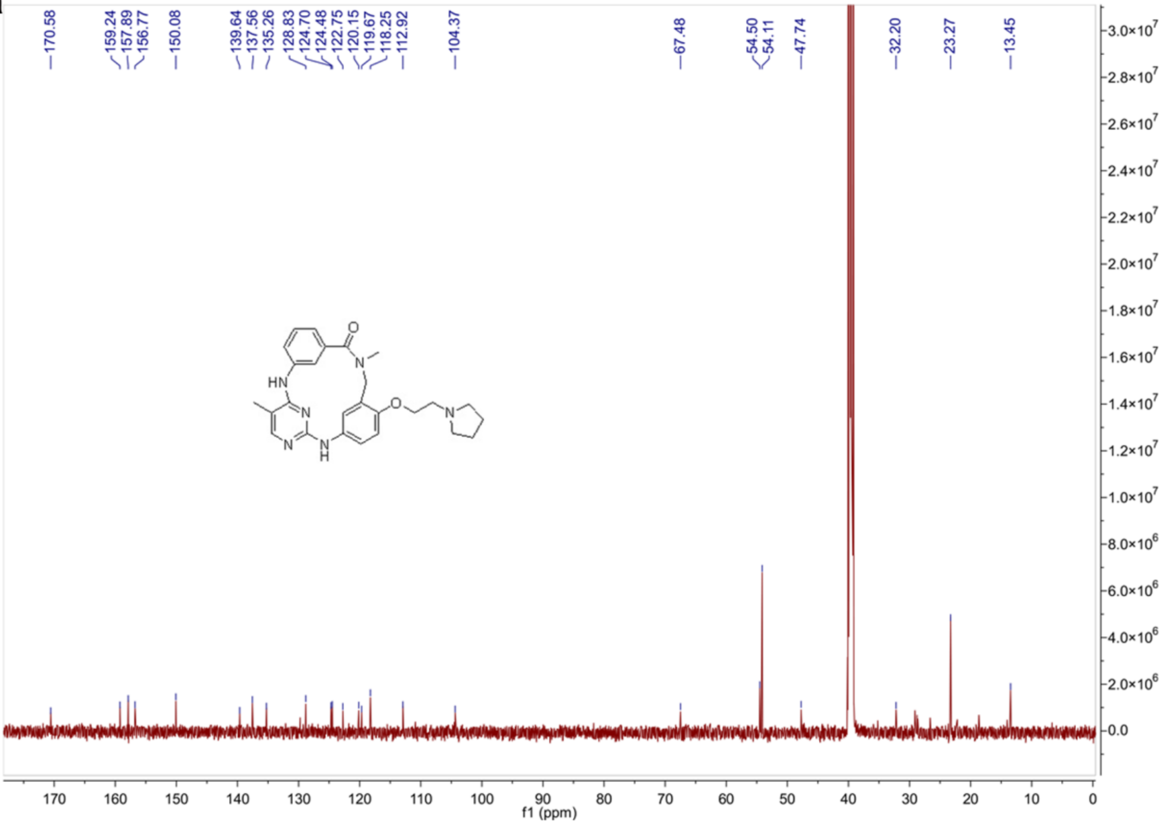

b

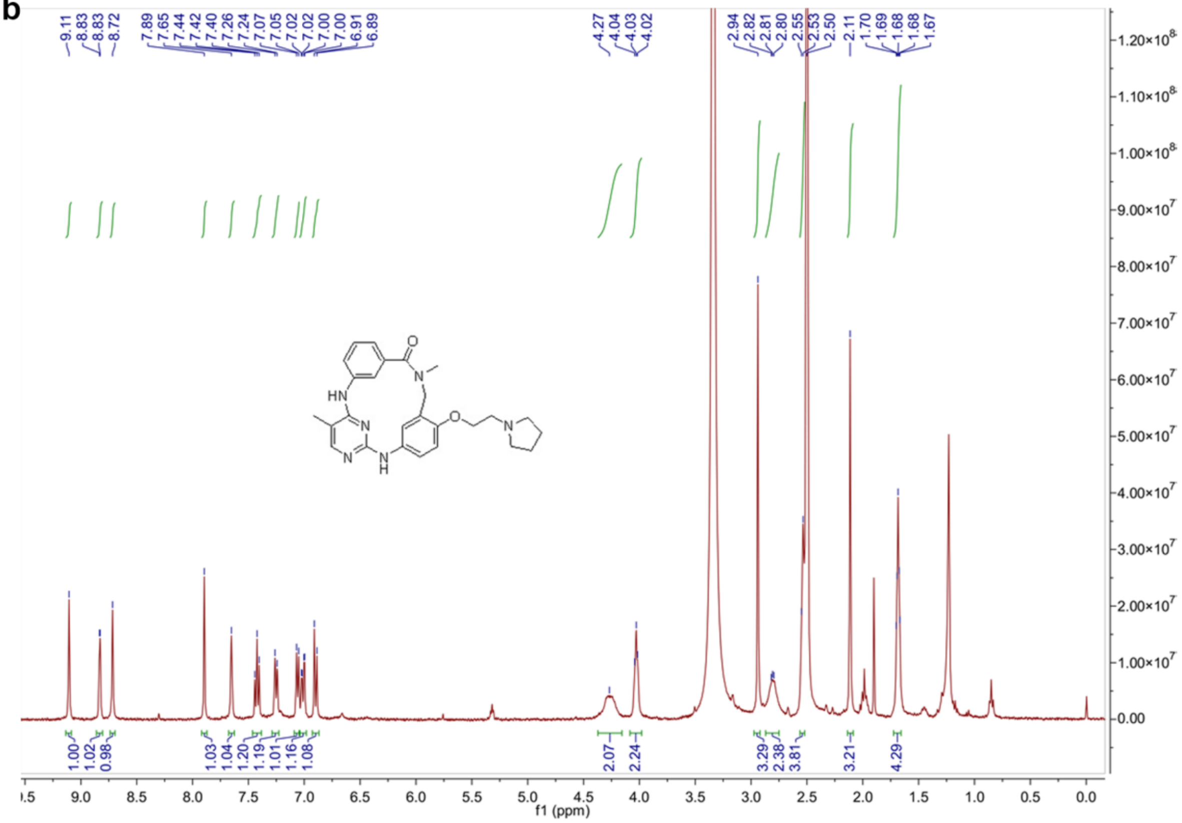

Supplementary Fig. 14. NMR spectrometry data of compound 2. a ^{13}C NMR spectrometry data, **b** ^{1}H NMR spectrometry data.

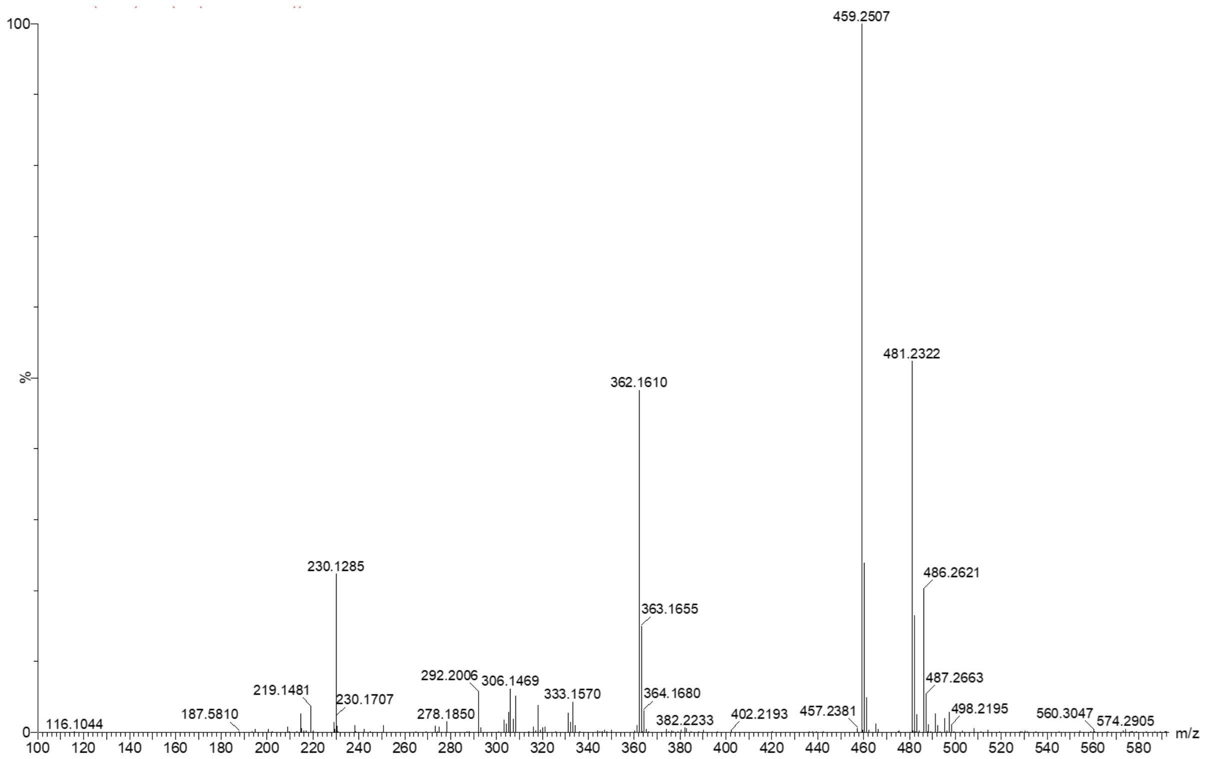

Supplementary Fig. 15. HRMS (ESI) spectrometry data of compound 2. $[M+H]^+$ calcd for $C_{26}H_{31}N_6O_2$ 459.2508; found 459.2507.

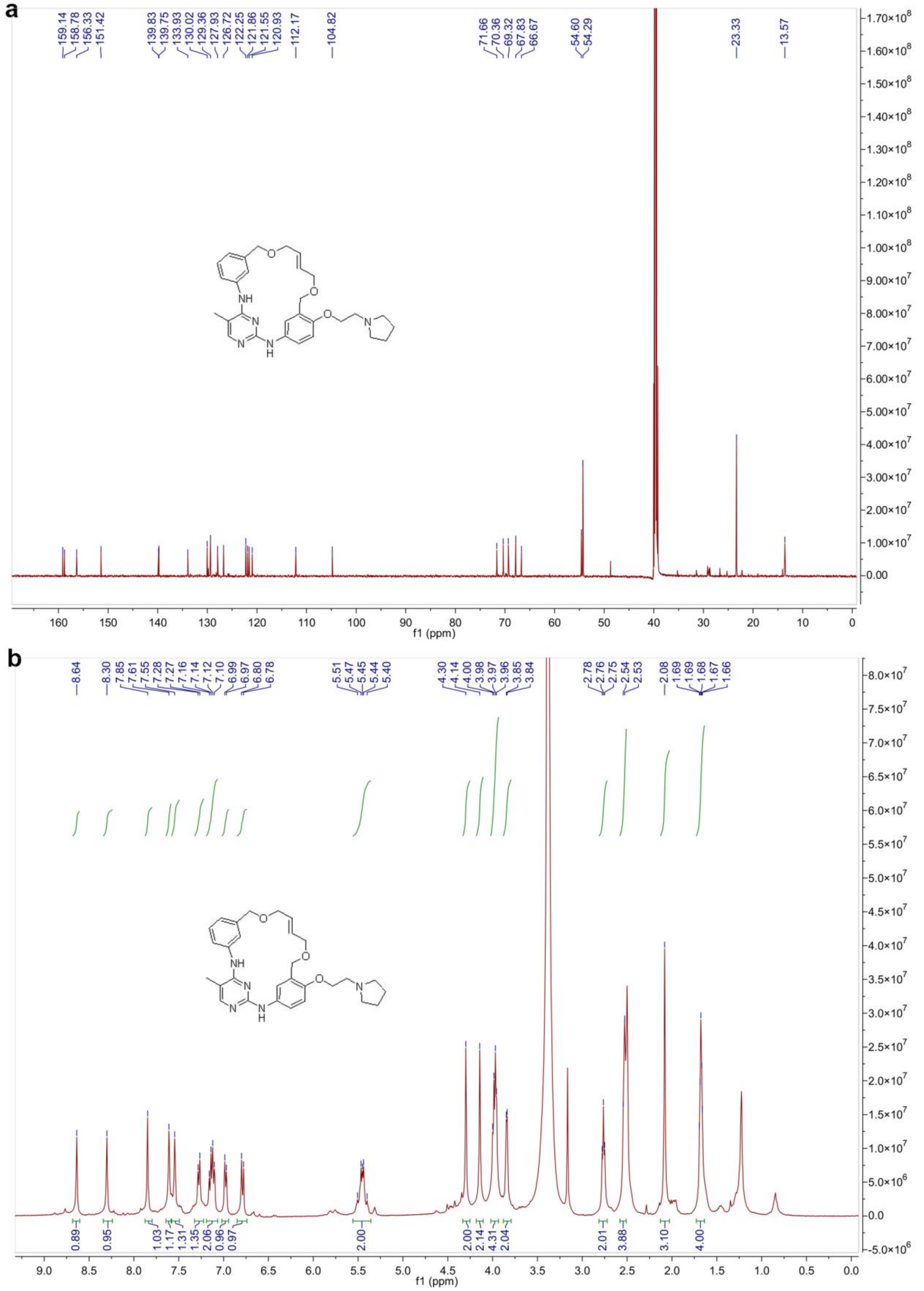

Supplementary Fig. 16. NMR spectrometry data of compound 3. a ^{13}C NMR spectrometry data, **b** ^{1}H NMR spectrometry data.

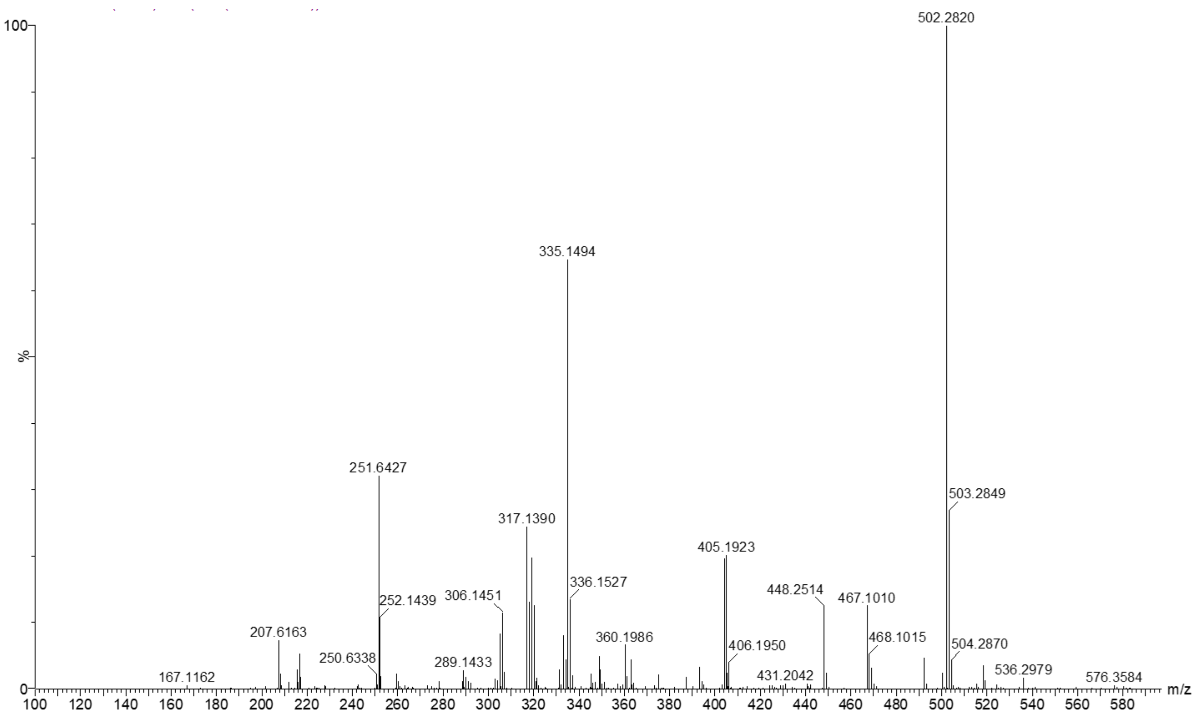

Supplementary Fig. 17. HRMS (ESI) spectrometry data of compound 3. [M+H]$^+$ calcd for $C_{29}H_{36}N_5O_3$ 502.2818; found 502.2820.
